# A Joint Low-Power Cell Search and Frequency Tracking Scheme in NB-IoT Systems for Green Internet of Things

**DOI:** 10.3390/s18103274

**Published:** 2018-09-29

**Authors:** Yu Li, Shuo Chen, Wenqiang Ye, Fujiang Lin

**Affiliations:** Department of Electronic Science and Technology, University of Science and Technology of China, Hefei 230027, China; csbao@mail.ustc.edu.cn (S.C.); lizard909@126.com (W.Y.); linfj@ustc.edu.cn (F.L.)

**Keywords:** Internet-of-Things, NB-IoT, cell search, NPSS, NSSS, frequency tracking

## Abstract

As a dedicated communication protocol for Internet-of-Things, narrowband internet of things (NB-IoT) needs to establish the communication link rapidly and reduce retransmissions as much as possible to achieve low power consumption and stable performance. To achieve these targets, the low-power scheme of the initial cell search and frequency tracking is investigated in this paper. The cell search process can be subdivided into narrowband primary synchronization signal (NPSS) detection and narrowband secondary synchronization signal (NSSS) detection. We present an NPSS detection method whose timing metric is composed of symbol-wise autocorrelation and a dedicated normalization factor. After the detection of NPSS, the symbol timing and fractional frequency offset estimation is implemented in a resource-efficient way. NSSS detection is conducted in the frequency domain with a calculation-reduced algorithm based on the features of NSSS sequences. To compensate the accumulated frequency offset during uplink transmission, a pilot-aided rapid frequency tracking algorithm is proposed. The simulation results of the proposed cell search scheme are outstanding in both normal coverage and extended coverage NB-IoT scenarios, and the accumulated frequency offset can be estimated with high efficiency.

## 1. Introduction

Nowadays, the interconnection of all things has become a major trend. Internet of things (IoT) is playing a key role in the fields of intelligent city, industry, agriculture and so on. According to the investigation of the International Energy Agency, there will be more than 14 billion IoT devices by 2020. With the aims of being green and reducing costs, these devices need to be connected to the Internet for remote control. Therefore, IoT technologies that fulfil these features are called Low Power Wide Area Network (LPWAN). There are two main categories of LPWAN technologies: technologies that operate in the unauthorized frequency spectrum, such as Lora, Sigfox, etc.; the existing protocols working with the cellular network, and Global System for Mobile Communication (GSM) and enhancements for Machine Type Communications (eMTC). The technologies working in the authorized frequency spectrum support every device to connect to the Internet independently, which makes the IoT networks more flexible. However, existing cellular network protocols can hardly support such a large number of IoT devices in the future.

To meet this urgent demand, the 5th generation wireless communication New Radio (5G NR) outlines the massive Machine Type Communication (mMTC) scenario [1]. Nevertheless, this machine-to-machine communication specification is scheduled to be released in the 3GPP Release-16 by the 3rd Generation Partnership Project (3GPP). For this reason, a pioneer air-interface, namely narrowband internet-of-things (NB-IoT), is supplemented to the Long-Term Evolution (LTE) which is complemented in 3GPP Release-14 [2]. As a specified machine type communication technology, NB-IoT not only boasts low energy consumption [3], but can also access the cellular network. This means that NB-IoT can be directly deployed in GSM, Universal Mobile Telecommunications System (UMTS) and LTE. NB-IoT has four primary characteristics: (1) In the same frequency band, NB-IoT gains 20 dB minimum coupling loss (MCL) compared with legacy LTE, so the coverage is expanded by 100 times theoretically. (2) The mass connection capacity allows one NB-IoT sector to support 100 thousand connections at most. (3) Low power consumption design guarantees that the standby time of NB-IoT devices can be up to 10 years. (4) By reducing cost, the price of a single NB-IoT module is expected to be no more than 5 US dollars.

The IoT scenarios deploying NB-IoT devices can gain special advantages. Hospitals, banks, and other sites that need high trustworthiness should consider privacy and security at first before deploying IoT services. NB-IoT has an innate feature to counteract this challenge: its spectrum is licensed. Moreover, the authors of [4] also consider a specific scenario and model the reliability of NB-IoT communication, and they give a trust-based solution with NB-IoT. Another area in need of IoT is the automobile field, including connection among cars and self-driving. Vitaly P. et al. [5] present an opportunistic crowdsensing scenario where traffic from a large number of connected sensors is transmitted over NB-IoT, and the results show that NB-IoT technology has the ability of sharing dynamic radio resources between vehicular base stations. In addition to the above, the positioning function supplemented into NB-IoT in Release-14 is also proved capable of offering reliable positioning function by Hu S. et al. in [6]; they also improve the positioning performance in NB-IoT with the observed-time-difference-of-arrival (OTDOA) algorithm.

Although NB-IoT has many advantages, considering that billions of NB-IoT devices will be deployed to every corner of the world, how to construct green IoT wireless communication networks with NB-IoT is a challenging issue. Since green IoT wireless communication networks are required to be built as economically and energy-efficient as possible, the massive NB-IoT terminals which make up the core parts of green IoT networks still need to be further optimized with low power consumption techniques. One significant majorization point is to optimize the process of the initial cell search and frequency tracking. More specifically, novel detection and synchronization methods should be presented to reduce the initial cell search and frequency tracking time in NB-IoT systems, and this operation shortens the working time of each transmission, which will extend the battery life of NB-IoT terminals.

In this paper, we investigate the efficient cell search and frequency tracking schemes to reduce the communication link setup time and retransmissions. Everything is double-edged: if we want to shorten the operation time, a certain amount of hardware resources and computation complexity will be sacrificed. Nevertheless, the increased cost introduced by the digital circuit resources is almost negligible, and the added power consumption caused by computation complexity is incomparable when compared with the RF transceiver. That is because the RF transceiver consumes most of the power of the whole transceiver. So, it is reasonable to exchange operation efficiency with computation complexity. In this way, low-power IoT networks are obtained. For NB-IoT cell search, robust performance and rapid estimation are two major objectives. The NB-IoT cell search process can be divided into narrowband primary synchronization signal (NPSS) detection and narrowband secondary synchronization signal (NSSS) detection. During the above procedures, coarse time of arrival (ToA) detection, timing synchronization and carrier frequency offset (CFO) recovery are also essential to ensure reliable cell ID detection. The MCL of NB-IoT can be 164 dB, which demands that NB-IoT terminals can build communication links with the base station at a very low signal-to-noise ratio (SNR). On the other hand, under economical considerations, a low precision oscillator is adopted by NB-IoT devices. For this reason, frequency tracking must be conducted during the transmission.

To describe the whole process and the problems encountered clearly, an overview of each procedure is presented. Firstly, when the downlink NB-IoT frames arrive, the NPSS detector will perform coarse ToA detection with the assistance of NPSS [7], and the detector is conventionally constructed with a timing metric function which is the ratio of sliding autocorrelation and a normalization factor. However, the NB-IoT needs to adapt a low SNR environment and large frequency offset, but the performance of the traditional timing metric function is seriously affected by noise and frequency offset. Secondly, after the coarse timing synchronization is completed, fine timing synchronization and frequency offset estimation will also be done in the time domain. Then, the NB-IoT cell ID is detected in the frequency domain. In legacy LTE, the cell ID is jointly determined by primary synchronization signal (PSS) and secondary synchronization signal (SSS) [2]. However, the cell ID of NB-IoT is decided by NSSS entirely, which has more diverse sequences compared with LTE [8]. Finally, when a stable communication link has been built up, frequency tracking is essential for NB-IoT systems because of the residual frequency offset after CFO recovery and the continuous frequency drift caused by low-cost oscillators.

In the proposed cell search scheme, the fractional frequency offset (FFO) pre-estimator and noise elimination circuit are applied to promote the performance of NPSS detection. The IFO is compensated with a maximum likelihood (ML) cross correlation method in the time domain. Additionally, considering the timing uncertainty introduced by the multi-path fading channel, fine timing offset is estimated jointly by the aforementioned ML solution. Then, the fast Fourier transform (FFT) transforms the time domain signals into frequency domain signals. NSSS detection is conducted under the premise that symbol timing synchronization and CFO recovery have been completed. Since the extended coverage of NB-IoT demands that the systems can tolerate very low SNR, e.g., −15 dB, a low computation complexity method is proposed to lessen the burden of NB-IoT terminals. Considering the diversity of NSSS sequences, divide-and-conquer is applied to accelerate the traversal search. When performing frequency tracking, NPSS, NSSS and NPBCH are jointly used to estimate the residual frequency offset. Related indicators and simulation results are presented to evaluate the performance of target designs.

The rest of this paper is organized as follows. Related work is discussed in Section 2. Section 3 illustrates the frame structure of NB-IoT. Problems of cell search and frequency tracking are formulated in Section 4. Section 5 presents the NB-IoT cell search method with the assistance of synchronization signals. The efficient frequency tracking procedure is suggested in Section 6 during the NB-IoT uplink transmission gap (UTG). We discuss the simulation results in Section 7. Finally, some conclusions and future work are given in Section 8.

## 2. Related Work

Both cell search and frequency tracking are important issues in NB-IoT and legacy LTE, and many articles on these points have been published by scholars. As the cell search procedure can be divided into NPSS detection and NSSS detection, we will discuss the existing solutions in three parts: (1) for NPSS detection, (2) for NSSS detection, and (3) for frequency tracking separately.

(1) Although NPSS detection is a problem of deterministic signal detection, the signal location is uncertain and it is actually a synchronization problem. Traditionally, NPSS detection is accomplished with symbol-wise sliding autocorrelation by using the duplicate property of NPSS [7], and this method tries to use quite several NB-IoT frames to achieve acceptable performance. However, to reduce the hardware consumption and computation complexity, this method operates at very low frequency with the decimated samples. As a result, lots of radio frames are occupied by the NPSS detector which increases the communication link setup time of the NB-IoT transceiver system and results in excessive power consumption. To shorten the detection time, Abdelmohsen A. and Walaa H. [8] adopt a full rate autocorrelation method. As they make the autocorrelation window longer than one subframe, the detected location of NPSS will become indistinct. In [9], Kroll H. et al. present an ML NPSS detector which can jointly estimate the frequency offset and symbol timing. The authors declare that this solution can estimate the whole range frequency and timing offset simultaneously, and it is true that it can deal with small range frequency offset. However, the estimation accuracy is severely restricted by the FFT points and working frequency. If high-precision frequency offset tracking is demanded, more than 64 k points FFT is demanded, which is unrealistic in NB-IoT terminals. Another aspect is that the existence of an integer frequency offset is not considered in this solution. Additionally, frame synchronization based on duplicated synchronization signals becomes the research object of many scholars. Timothy M. Schmidl and Donald C. Cox [10] proposed taking advantage of the preamble to construct a timing metric (TM) which consists of an autocorrelation part and a normalization factor (NF). To ensure that the TM is robust to CFO, a modified TM is proposed in [11]. To deal with different CFO and SNR scenes [12], summarizes two TMs based on two differential NFs and gives the suitable CFO and SNR ranges for each TM. The results show that these methods reduce the ability to deal with low SNR conditions. The authors of [13,14,15] make use of high-order statistics to accelerate the detection and synchronization procedures. Nevertheless, even though these methods bring some performance improvement, the enormous amount of calculation is difficult to achieve with the hardware of IoT terminals.
(2) When NPSS detection is conducted, and timing synchronization and CFO recovery have been completed, NSSS detection will be performed in the frequency domain. Different from legacy LTE, the cell ID of NB-IoT is decided by NSSS entirely which has more diverse sequences compared with LTE [8]. However, as both SSS detection and NSSS detection are determinate signal detection problems, many SSS detection schemes can be used for reference. Furthermore, these solutions can be mainly divided into two classes: coherent detection and non-coherent detection [16]. Considering the coherent SSS detection, the key operation is resorting to the known PSS to estimate the channel frequency responses (CFR) of each subcarrier in the frequency domain, and using the CFRs to improve the performance of correct SSS cross correlation results [17,18] . Unfortunately, despite NB-IoT operating in slow fading wireless environments [19,20], which means that even NPSS and NSSS are separated by no less than 4 subframes, the channels can still be regarded as constant. However, the NB-IoT terminals will not assume that NPSS and NSSS are transmitted on the same antenna port and the CFRs of NPSS may be different from NSSS [2]. The non-coherent method can be subdivided into differential correlation-based and partial correlation-based algorithms. Multifarious differential solutions have been proposed to accomplish the detection, for example, Zheng Du and Jinkang Zhu [21] adopt this method to reduce the effect of the dispersive channel. Nevertheless, the differential correlation would deteriorate SNR with the autocorrelation of received signals. The idea of partial correlation can be found in [22,23]. However, since NPSS has no repeatability among signals, this method is invalid in NB-IoT application. In addition to the above-mentioned methods, the authors of [24] search the correct SSS sequence by minimizing the Euclidian distance which is the basic application of ML detection and the performance will be reduced in the context of low SNR.
(3) A communication link would be set up as soon as the initial cell search has been completed. However, because of the residual frequency offset after CFO recovery and the continuous frequency drift caused by the oscillator, frequency offset tracking is essential for NB-IoT systems. Besides, one uplink transmission feature of NB-IoT is that a 40 ms downlink gap must be inserted between two 256 ms uplink transmission time units [2]. This is because the frequency drift value may exceed the tolerance of the synchronization circuits in base stations [25]. Typically, LTE frequency tracking algorithms mainly employ the references signal (RS) as auxiliary data. In some OFDM systems, due to multiple crystals being used, residual carrier frequency offset and sampling clock frequency offset need to be estimated separately [26]. This is impractical for NB-IoT terminals with the consideration of cost constraints. The authors of [27] adopt the autocorrelation of two identical symbols in the same subcarrier to calculate phase difference. The fatal drawback is that the number of repetitive preamble pairs may be inadequate to achieve adequate frequency tracking accuracy at low SNRs. By using the cross correlation process, the solution derived in [28] can make use of different pilots. Two extra multiplications are needed in the calculation of each correlation pair. Considering the frequency estimation precision demanded by the NB-IoT system, the ML frequency estimation solution proposed in [9] is unsuitable for frequency tracking. That is because when the tracking accuracy is 40 Hz, the number of FFT points should be no less than eight times of 1024.

## 3. NB-IoT Frame Structure

The downlink transmission of NB-IoT is similar to legacy LTE with 15 KHz subcarrier bandwidth. However, only one physical resource block (PRB) [2] is occupied by NB-IoT. Since 12 subcarriers form one PRB, the downlink bandwidth of NB-IoT is 200 KHz, including 20 KHz for the guard band. In the time domain, the length of one radio frame is 10 ms, and a radio frame consists of 10 subframes. Then, a subframe is subdivided into two slots and each slot contains seven OFDM symbols. Besides, only normal cyclic profix (CP) is supported by NB-IoT. There are three deployment modes for NB-IoT, namely in-band, guard-band and standalone. In-band operation means that the NB-IoT band locates within legacy LTE, and the time and frequency resources reserved for LTE will not transmit NB-IoT signals. The guard-band mode indicates that the NB-IoT carrier is deployed in the guard band of the LTE carrier. As for standalone operation, it is realized by replacing GSM carriers with NB-IoT carriers. There are three physical channels and three physical signals defined in [2]. They are listed as follows:

- Narrowband physical downlink shared channel (NPDSCH).
- Narrowband physical broadcast channel (NPBCH).
- Narrowband physical downlink control channel (NPDCCH).
- Narrowband reference signal (NRS).
- Narrowband synchronization signal: NPSS and NSSS.
- Narrowband positioning reference signal (NPRS).

**Figure 1:**
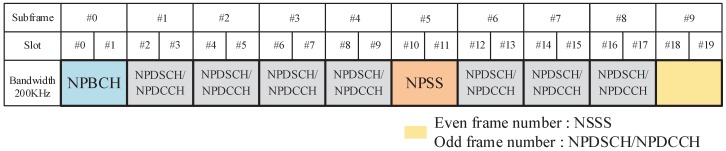
Diagram of the distribution of physical channels and physical signals in one NB-IoT radio frame.

Figure 1 illustrates the distribution of the physical channels and signals in NB-IoT radio frames. It is worth mentioning that NSSS only appears on the 9th subframe of even radio frames.

### 3.1. NPSS Sequence

The signals of NPSS occupy 11 symbols in subframe 5 of every radio frame. To achieve excellent correlation property, the Zadoff–Chu frequency domain is used to generate the NPSS sequence. The sequence in each NPSS symbol is identical and the only difference is the code cover which gives a steep rolloff timing metric to the NPSS detector. The complex exponential expression of the NPSS sequence is shown as follows

(1) $$a\left(k\right)=e^{-j\frac{\pi 5k(k+1)}{11}},k=0,1,2\ldots 10.$$

The code cover is a defined zero-one sequence, and Table 1 gives the exact values.

**Figure 2:**
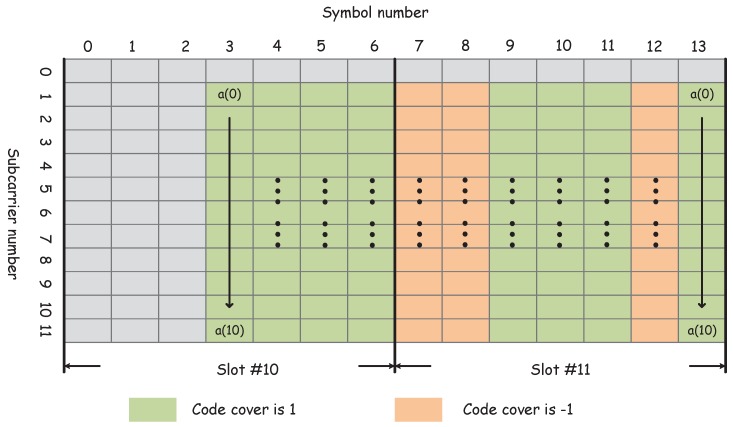
Diagram of the mapping of NPSS resource elements in slots 10 and 11. The time region starts from symbol 3 to 13, and the frequency region starts from subcarrier 1 to 11.

The NPSS subframe structure is illustrated in Figure 2. Each time domain symbol is obtained by multiplexing the inverse fast Fourier transform (IFFT) of a(k) with code cover.

### 3.2. NSSS Sequence

The NSSS is constructed by multi-parameter frequency domain Zadoff–Chu sequences. The parameters contain cell ID, radio frame number and their derived parameters. There are 2016 different NSSS sequences in all, which makes the computation complexity of NB-IoT cell ID identification much higher than LTE. Equation (2) shows the formulation of NSSS,

(2) $$\begin{array}{cc} b(n) & =g_{q}\left(m\right)e^{-j2\pi \theta_{f}n}e^{-j\frac{\pi un^{\prime }\left(n^{\prime }+1\right)}{131}}\\ n & =0,1,\ldots ,131. \end{array}$$

Table 2 describes the derivation of the parameters in the above equation. $r_{f}$ represents the frame number. Because NSSS can only be mapped on even frames, the frame number needs to fulfil $r_{f}\;\mathrm{mod}\;2=0$. Furthermore, $N_{ID}^{Ncell}$ denotes the cell ID. As NB-IoT is a multi-cell communication system, the total number of cell IDs can be as high as 504. Moreover, $g_{q}$ consists of four binary sequences defined in [2].

### 3.3. Narrowband Reference Signal

As defined in NB-IoT specification [2], NRS could be transmitted on one or two antenna ports. The NRS sequence generation process is similar to that of LTE and the mapped NRS in the frequency domain with different numbers of antenna is described in [7]. The distance between two NRS symbols on the same subcarrier is one slot. When two antenna ports are used, the resource elements of NRS in another antenna port must be kept unused within the radio frames from the current antenna.

### 3.4. NPBCH

NPBCH is transmitted in the subframe 0 of every radio frame and the first three symbols are unused. The data of NPBCH is identical in eight consecutive radio frames. In in-band mode operation, the LTE cell-specific reference signal (CRS) can also be deployed in the NPBCH subframe. So, in addition to NPBCH data, CRS, NRS and channel control signals can also be included in NPBCH subframes.

## 4. Signal Models

NB-IoT is designed to operate in half-duplex Frequency-Division Duplexing (FDD) mode with normal CP. The frequency domain data $S_{p,r_{f}}^{\left(m\right)}\left(k\right)$ carried by the *k*th subcarrier of NB-IoT is symmetrically distributed on the 200 KHz bandwidth with a 10 KHz guard band in each side and the central frequency point is unused to avoid any problems caused by direct current (DC). After conducting IFFT and adding normal CP, the time domain NB-IoT radio frame samples $s_{p,r_{f}}^{\left(m\right)}\left(n\right)$ are obtained. p∈[0,P] is the subframe index within radio frame $r_{f}$. m∈[0,M] represents the OFDM symbol index in one subframe, and $n\in [1,N+N_{g}]$ denotes the sample index in a OFDM symbol. Here, *N* is the IFFT size and $N_{g}$ is the length of normal CP. Besides, there are relationships of P=9 and M=13 in the NB-IoT system.

Then, the signals transmitted from the base station are propagated by the multi-path fading channel of Extended Typical Urban (ETU) or Extended Pedestrian A (EPA), and the route mean square delay spread (RMS-DS) and coherence bandwidth of ETU and EPA are 991 ns and 45 ns, and 1.01 MHz and 22.2 MHz, respectively. The radio signals are received by the NB-IoT radio frequency receiver which down converts the radio signals to the baseband. After that, the in-phase and quadrature vectors of analog baseband signals are quantified by ADCs. Considering the additive white Gaussian noise (AWGN), ETU or EPA multi-path channels and the CFO introduced by oscillators in the transmitter and receiver, the samples of the received signals can be expressed as follows:

(3) $$r_{p,r_{f}}^{\left(m\right)}\left(n\right)=e^{j2\pi \frac{\left(n+m\left(N+N_{g}\right)\right)\left(\varepsilon_{F}+\varepsilon_{I}\right)}{N}}\sum_{l=0}^{L-1}h\left(l\right)s_{p,r_{f}}^{\left(m\right)}\left(l-n\right)+\omega \left(n\right),$$

where h(l) is the channel impulse response of the *l*th frequency selective fading channel, and *L* is the taps of the fading channel. $\varepsilon_{F}$ represents the FFO, and the IFO part is denoted by $\varepsilon_{I}$. ω is the zero mean AWGN introduced by the communication link with variance of $\sigma_{\omega }^{2}$. In the working scenarios of NB-IoT, the maximum frequency offset range may achieve [−25.5 KHz, 25.5 KHz] [29]. The received NPSS symbols are maintained identical by the following relationship:

(4) $$c\left(0\right)r_{5,r_{f}}^{\left(3\right)}\left(n\right)=c\left(1\right)r_{5,r_{f}}^{\left(4\right)}\left(n\right)=\cdots =c\left(M-3\right)r_{5,r_{f}}^{\left(M\right)}\left(n\right).$$

**Figure 3:**
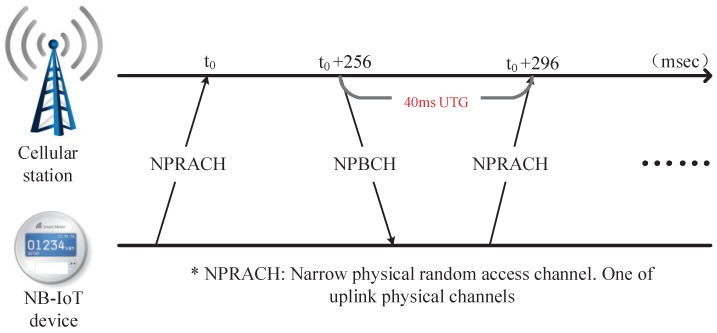
Illustration of the UTG inserted during the uplink transmission of NPRACH.

Traditionally, the time domain symbol synchronization with identical preambles is accomplished with an autocorrelation timing metric (TM) which consists of an autocorrelation part and a normalization factor. The following equation indicates the basic implementation [10].

(5) $$\gamma \left(\tau \right)=\frac{\alpha (\tau )}{\beta (\tau )}.$$

with

(6) $$\alpha \left(\tau \right)={\left|\sum_{n=1}^{N}\sum_{m=3}^{M-1}c\left(m-3\right)r_{p,r_{f}}^{\left(m\right)}\left(n\right)c\left(m-2\right)r_{p,r_{f}}^{(m+1)*}\left(n\right)\right|}^{2}.$$

(7) $$\beta \left(\tau \right)=\sum_{n=1}^{N}\sum_{m=3}^{M}|r_{p,r_{f}}^{\left(m\right)}\left(n\right){|}^{2}.$$

where τ represents the start time of the detection window, and ${\left(\cdot \right)}^{*}$ is complex conjugation. However, when in the context of low SNR, the conventional normalization factor (NF) in Equation (7) will be invalid. So, an effective NF is urgently needed to improve the performance of the NPSS detector. In the process of cell ID identification, the detection of NSSS from numerous sequences runs counter to the concept of low complexity design of NB-IoT terminals. Furthermore, for the reason that the coherent algorithms for SSS detection are inapplicable for NSSS, a suitable correlation detection method is urgently needed. Another significant feature of NB-IoT is that a 40 ms UTG should be inserted into the uplink transmission once the frequency offset exceeds the predefined threshold [2], and the specific implementation is shown in Figure 3.

During UTG, the system converts to downlink transmission to estimate and compensate the residual frequency offset (RFO) [30,31], and the initial cell search and coarse synchronization are considered to have been accomplished. Then, the frequency domain signals from FFT can be defined as $R_{p,r_{f}}^{m}\left(k\right)$, and have the formulation of

(8) $$R_{p,r_{f}}^{\left(m\right)}\left(k\right)=\eta \left(\varepsilon_{RFO}\right)H_{p,r_{f}}^{\left(m\right)}\left(k\right)S_{p,r_{f}}^{\left(m\right)}\left(k\right)e^{j2\pi \frac{m\left(N+N_{g}\right)\varepsilon_{RFO}}{N}}+\Omega \left(k\right),$$

where $H_{p,r_{f}}^{\left(m\right)}\left(k\right)$ and Ω(k) denote the CFR and the noise of the *k*th subcarrier separately. $\varepsilon_{RFO}$ is the normalized RFO. The constant amplitude and phase deviation caused by RFO are represented by $\eta (\varepsilon_{RFO})$ which is illustrated in [32]. Considering that the low precise digital controlled crystal oscillators are used by NB-IoT terminals, the challenging problem is how to reduce the RFO to a tolerable scope of uplink transmission within the UTG.

## 5. NB-IoT Cell Search

In this section, the proposed cell search method is presented, which is accomplished in both the time domain and frequency domain. The time domain process includes NPSS detection and time domain synchronization, and the cell ID identification is completed in the frequency domain.

### 5.1. NPSS Detection and Time Domain Synchronization

Constructing a proper TM is the most efficient way to detect NPSS. However, because the traditional NF in TM will be invalid in the context of low SNR [30] and the SNR of extended coverage NB-IoT can be −15 dB, solutions in [7,8] abandon the use of NF. Nevertheless, this compromise reduces the consumption of some hardware resources, and the lengthened synchronization time consumes much power which is vital for NB-IoT terminals. Here, a noise-eliminated NF with an FFO pre-estimator is proposed to deal with low SNR conditions. As the NB-IoT samples are preceded by noise samples and NB-IoT terminals work with low mobility, the noise power $\sigma_{\omega }^{2}$ can be estimated by these noise-only samples. The calculation process of autocorrelation summation can apply the sliding method in [10]. The application of code cover in [11] avoids the plateau in the presence of NPSS, and a steep roll-off trajectory will present. As for the IFO and fine timing error, the cross-correlation between coarse detected NPSS and ideal NPSS is used as an estimator.

In the following, the detailed procedures are discussed. As shown in Equation (5), the correct time of NPSS is given by

(9) $$\tau_{0}=\underset{\tau }{\arg\max}\left|\gamma \left(\tau \right)\right|,$$

and the TM proposed in this paper is defined as $\gamma_{prop}\left(\tau \right)$. The symbol-wise autocorrelation part and the noise-eliminated NF are represented by $\alpha_{SW}$ and $\beta_{NE}$, respectively. To reduce detection complexity, the authors of [7] accomplish the NPSS detection process at quite low frequency. However, not only do more radio frames have to be used, but also the synchronization precision is decreased. To accelerate this procedure, the authors of [8] adopt the octuple working frequency of [7]. The autocorrelation distance in this method is only one OFDM symbol. Our scheme is implemented with a simplified symbol-wise method compared with [7] in order to adapt to the higher working frequency. Thus, $\alpha_{SW}$ has the following form.

(10) $$\alpha_{SW}\left(\tau \right)={\left|\sum_{r_{f}^{\prime }=1}^{R_{f}^{\prime }}\sum_{v=1}^{V}\sum_{m^{\prime }=3}^{M^{\prime }-v}\sum_{n=1}^{N+N_{g}}c\left(m^{\prime }-3\right)r_{p,r_{f}^{\prime }}^{(m^{\prime })*}\left(n\right)c\left(m^{\prime }-3+v\right)r_{p,r_{f}^{\prime }}^{\left(m^{\prime }+v\right)}\left(n\right)\right|}^{2},$$

where τ denotes the start sample of the subframe *p* in radio frame $r_{f}^{\prime }$, and ${\left(\cdot \right)}^{\prime }$ is the hypothetical value of each parameter. *v* represents the number of OFDM symbols between the two parts of one correlation pair. Furthermore, the calculation of $\alpha_{SW}\left(\tau +1\right)$ can be conducted in a convenient way,

(11) $$\begin{array}{cc} \alpha_{SW}\left(\tau +1\right)= & |\sum_{r_{f}^{\prime }=1}^{R_{f}^{\prime }}\sum_{v=1}^{V}\sum_{m^{\prime }=3}^{M^{\prime }-v}(\sum_{n=1}^{N+N_{g}}c\left(m^{\prime }-3\right)r_{p,r_{f}^{\prime }}^{(m^{\prime })*}\left(n\right)c\left(m^{\prime }-3+v\right)r_{p,r_{f}^{\prime }}^{\left(m^{\prime }+v\right)}\left(n\right)\\ & -c\left(m^{\prime }-3\right)r_{p,r_{f}^{\prime }}^{(m^{\prime })*}\left(1\right)c\left(m^{\prime }-3+v\right)r_{p,r_{f}^{\prime }}^{\left(m^{\prime }+v\right)}\left(1\right)\\ & +c\left(m^{\prime }-3\right)r_{p,r_{f}^{\prime }}^{(m^{\prime })*}\left(N+N_{g}+1\right)c\left(m^{\prime }-3+v\right)r_{p,r_{f}^{\prime }}^{\left(m^{\prime }+1\right)}\left(N+N_{g}+v\right)){|}^{2}. \end{array}$$

As the NB-IoT terminals are considered to be working with low mobility, the number of used radio frames $R_{f}^{\prime }$ and the maximum symbol spacing *V* can be adjusted according to the SNR.

**Figure 4:**
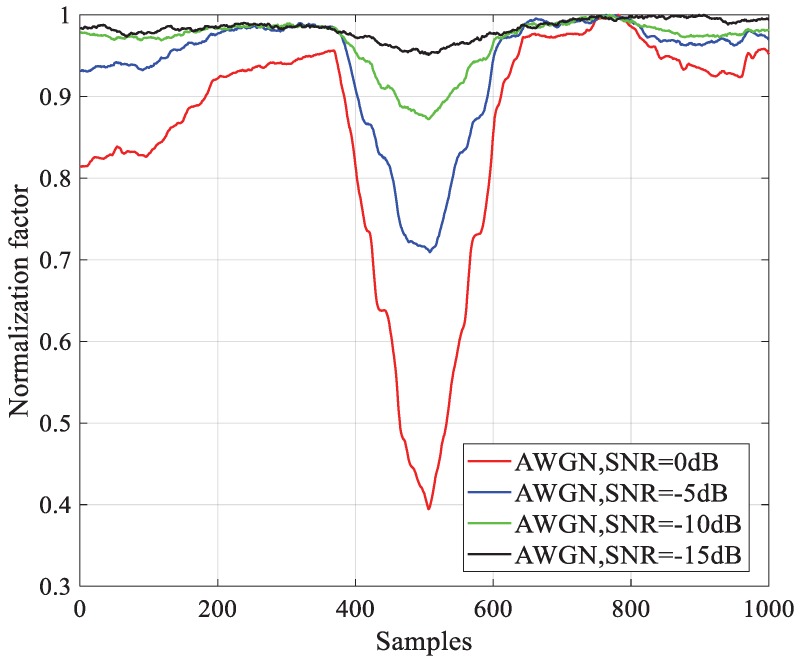
Comparison of MOD normalization factors at different SNRs.

All the NPSS detection methods implemented with TM no longer use NF. That is because NF is almost ineffective in low SNR conditions, as shown in Figure 4, where we use the Magnitude-of-Difference (MoD) NF proposed in [12] which has good performance in the AWGN channel. The result shows that although the NF still contributes a valley to add to the resolution of TM at the correct time, the valley decreases fast with the SNR. One feasible solution is to weaken the influence of noise on NF. To achieve this aim, we created a noise-eliminated differential NF which is composed of a differential part and a noise elimination part. The noise power $\sigma_{\omega }^{2}$ can be estimated with a moving average filter (MAF) before the arrival of the NB-IoT frame. The average window is set as $T_{0}$, and the estimation is as follows,

(12) $$\sigma_{\omega }^{2}\left(n_{0}\right)=\frac{1}{T}\sum_{n=n_{0}}^{n_{0}+T}{\left|r_{\omega }\left(n\right)\right|}^{2},$$

where $n_{0}$ denotes the window start time of MAF. Furthermore, in order to promote the accuracy of estimation, Λ consecutive windows are coherently averaged until the NB-IoT radio frame presents, and an infinite impulse response filter is applied.

(13) $${\bar{\sigma }}_{\omega }^{2}\left(n_{\lambda }\right)=\eta {\bar{\sigma }}_{\omega }^{2}\left(n_{\lambda -1}\right)+\left(1-\eta \right)\sigma_{\omega }^{2}\left(n_{\lambda }\right),$$

with

(14) $${\bar{\sigma }}_{\omega }^{2}\left(n_{0}\right)=\sigma_{\omega }^{2}\left(n_{0}\right),$$

here, η is set as a constant 0.8 (This value is obtained by software simulation with proper NB-IoT working environment). Conventional MoD NF has the original form of

(15) $$\beta_{MOD}\left(\tau \right)=\sum_{n=\tau +1}^{\tau +N+N_{g}}{\left|r\left(n\right)-r\left(n+N+N_{g}\right)\right|}^{2}.$$

The above equation is the two identical parts version in [11]. However, to adapt to the structure of NPSS, we modify it into a symbol-wise form similar to $\alpha_{SW}\left(\tau \right)$ as the following equation.

(16) $$\beta_{MOD}^{\prime }\left(\tau \right)=\sum_{r_{f}^{\prime }=1}^{R_{f}^{\prime }}\sum_{v=1}^{V}\sum_{m^{\prime }=3}^{M^{\prime }-v}\sum_{n=1}^{N+N_{g}}{\left|c\left(m^{\prime }-3\right)r_{p,r_{f}^{\prime }}^{\left(m^{\prime }\right)}\left(n\right)-c\left(m^{\prime }-3+v\right)r_{p,r_{f}^{\prime }}^{\left(m^{\prime }+v\right)}\left(n\right)\right|}^{2}.$$

Since the NB-IoT terminals work with a slow fading channel, the multi-path signals in one detection window can be assumed as independent zero mean Gaussian random signals with the variance of $\delta_{s}^{2}$. By using the approximation similar to [11] and considering the presence of NPSS, the below expression can be obtained.

(17) $$\begin{array}{cc} \beta_{MOD}^{\prime }\left(\tau_{p}\right) & \approx \sum_{v=1}^{V}R_{f}^{\prime }\left(N+N_{g}\right)\left(M^{\prime }-v-2\right)\left(\delta_{s}^{2}{\left|1-e^{j2\pi \frac{v\left(N+N_{g}\right)\varepsilon_{F}}{N}}\right|}^{2}+2\delta_{\omega }^{2}\right)\\ & =\kappa \left(v\right)\delta_{s}^{2}{\left|1-e^{j2\pi \frac{v\left(N+N_{g}\right)\varepsilon_{F}}{N}}\right|}^{2}+2\kappa \left(v\right)\delta_{\omega }^{2}, \end{array}$$

with

(18) $$\kappa \left(v\right)=\sum_{v=1}^{V}R_{f}^{\prime }\left(N+N_{g}\right)\left(M^{\prime }-v-2\right).$$

Similarly, when the NPSS is absent, the approximation is

(19) $$\beta_{MOD}^{\prime }\left(\tau_{a}\right)\approx 2\kappa \left(v\right)\left(\delta_{s}^{2}+\delta_{\omega }^{2}\right),$$

where $\tau_{p}$ and $\tau_{a}$ represent the present and absent time of the NPSS signal, respectively. The ratio between $\beta (\tau_{p})$ and $\beta (\tau_{a})$ dictates the performance of NF. Considering the following relationship

(20) $$ℜ=\frac{\beta_{MOD}^{\prime }\left(\tau_{p}\right)}{\beta_{MOD}^{\prime }\left(\tau_{a}\right)}=\frac{\kappa \left(v\right){\left|1-e^{j2\pi \frac{v\left(N+N_{g}\right)\varepsilon_{F}}{N}}\right|}^{2}+2\kappa \left(v\right)\frac{1}{SNR}}{2\kappa \left(v\right)\left(1+\frac{1}{SNR}\right)},$$

when an NF is effective, the parameter ℜ>0 is required. The precise range of $\varepsilon_{F}$ to fulfil ℜ>0 is too complex to be decided, as it is determined jointly by *V* and $M^{\prime }$. So, we investigate a reduced range by the following equation,

(21) $${\left|1-e^{j2\pi \frac{V\left(N+N_{g}\right)\varepsilon_{F}}{N}}\right|}^{2}<2,$$

with

(22) $$-\frac{N}{4V(N+N_{g})}<\varepsilon_{F}<\frac{N}{4V(N+N_{g})}.$$

Unfortunately, the $\varepsilon_{F}$ of NB-IoT can be any value in $\left[-\frac{N}{2(N+N_{g})},\frac{N}{2(N+N_{g})}\right]$. To lessen the $\varepsilon_{F}$ into the target range, a FFO pre-estimator is introduced. In the following, we will illustrate the implementation of the pre-estimator; then, its application will also be discussed.

Considering Equations (3) and (4), and using the intermediate calculation results of Equation (10), the FFO estimation at *v* can be expressed as

(23) $$\begin{array}{cc} {\hat{\varepsilon }}_{F}\left(v\right)= & \frac{N}{2\pi (N+N_{g})}\arg\left\{\sum_{r_{f}^{\prime }=1}^{R_{f}^{\prime }}\sum_{m^{\prime }=3}^{M^{\prime }-v}\sum_{n=1}^{N+N_{g}}c\left(m^{\prime }-3\right)r_{p,r_{f}^{\prime }}^{(m^{\prime })*}\left(n\right)c\left(m^{\prime }-3+v\right)r_{p,r_{f}^{\prime }}^{\left(m^{\prime }+v\right)}\left(n\right)\right\}\\ & -\pi <\arg\left\{\cdot \right\}<\pi , \end{array}$$

(24) $${\hat{\varepsilon }}_{F}=w_{1}{\hat{\varepsilon }}_{F}\left(1\right)+\sum_{v=2}^{V}w_{v}\Delta {\hat{\varepsilon }}_{F}\left(v\right),$$

with

(25) $$\Delta {\hat{\varepsilon }}_{F}\left(v\right)=\left\{\begin{array}{cc} {\hat{\varepsilon }}_{F}\left(v\right)-{\hat{\varepsilon }}_{F}\left(v-1\right)+\frac{N}{\left(N+N_{g}\right)}, & \mathrm{if}\;{\hat{\varepsilon }}_{F}\left(v\right)-{\hat{\varepsilon }}_{F}\left(v-1\right)<-\frac{N}{2(N+N_{g})}\\ {\hat{\varepsilon }}_{F}\left(v\right)-{\hat{\varepsilon }}_{F}\left(v-1\right)-\frac{N}{\left(N+N_{g}\right)}, & \mathrm{if}\;{\hat{\varepsilon }}_{F}\left(v\right)-{\hat{\varepsilon }}_{F}\left(v-1\right)>\frac{N}{2(N+N_{g})}\\ {\hat{\varepsilon }}_{F}\left(v\right)-{\hat{\varepsilon }}_{F}\left(v-1\right), & \mathrm{otherwise}, \end{array}\right.$$

where the values of weight parameters $w_{v}$ can be set as follows.

(26) $$w_{v}=\left\{\begin{array}{cc} \frac{1}{2^{v}}, & \mathrm{if}\;v=1,2,\cdots ,V-1\\ \frac{1}{2^{\left(v-1\right)}}, & \mathrm{if}\;v=V. \end{array}\right.$$

Another problem that needs to be considered is that the FFO should be compensated before the calculation of $\beta_{MOD}^{\prime }\left(\tau \right)$. ${\hat{\varepsilon }}_{F}$ can only be reliably estimated at the end of the detection window. A compromise solution is to modify Equation (16) into the following formulation.

(27) $$\begin{array}{cc} \beta_{MMOD}^{\prime }\left(\tau \right) & =\sum_{v=1}^{V}{\left|\sum_{r_{f}^{\prime }=1}^{R_{f}^{\prime }}\sum_{m^{\prime }=3}^{M^{\prime }-v}\sum_{n=1}^{N+N_{g}}c\left(m^{\prime }-3\right)r_{p,r_{f}^{\prime }}^{\left(m^{\prime }\right)}\left(n\right)-\sum_{r_{f}^{\prime }=1}^{R_{f}^{\prime }}\sum_{m^{\prime }=3}^{M^{\prime }-v}\sum_{n=1}^{N+N_{g}}c\left(m^{\prime }-3+v\right)r_{p,r_{f}^{\prime }}^{\left(m^{\prime }+v\right)}\left(n\right)\right|}^{2}\\ & =\sum_{v=1}^{V}{\left|A(v)-B(v)\right|}^{2}. \end{array}$$

Both A(v) and B(v) can be calculated separately during the detection window. After that, the estimated FFO is applied.

(28) $$\beta_{MMOD}^{\prime \prime }\left(\tau \right)=\sum_{v=1}^{V}{\left|A\left(v\right)-B\left(v\right)e^{-j2\pi \frac{v\left(N+N_{g}\right)\hat{\varepsilon_{F}}}{N}}\right|}^{2}.$$

It is obvious that when the NPSS is absent, the ${\hat{\varepsilon }}_{F}$ is just a random phase rotation to the NF. When the FFO estimation error fulfils the following relationship

(29) $$\left|\varepsilon_{F}-{\hat{\varepsilon }}_{F}\right|<\frac{N}{4V(N+N_{g})},$$

the *ℜ* of $\beta_{MMOD}^{\prime \prime }\left(\tau \right)$ can be written as

(30) $$\begin{array}{cc} ℜ=\frac{\beta_{MMOD}^{\prime \prime }\left(\tau_{p}\right)}{\beta_{MMOD}^{{}^{\prime \prime }}\left(\tau_{a}\right)} & =\frac{\kappa \left(v\right){\left|1-e^{j2\pi \frac{v\left(N+N_{g}\right)\left(\varepsilon_{F}-{\hat{\varepsilon }}_{F}\right)}{N}}\right|}^{2}+2\kappa \left(v\right)\frac{1}{SNR}}{2\kappa \left(v\right)\left(1+\frac{1}{SNR}\right)}\\ & >\frac{\kappa \left(v\right){\left|1-e^{j2\pi \frac{v\left(N+N_{g}\right)\left(\varepsilon_{F}-{\hat{\varepsilon }}_{F}\right)}{N}}\right|}^{2}}{2\kappa (v)}. \end{array}$$

The above equation shows that the SNR is larger, and the NF performance is better. Based on this analysis, the estimated noise power in Equation (13) can be applied to improve the proposed NF.

(31) $$\beta_{NE}\left(\tau \right)=\sum_{v=1}^{V}{\left|A\left(v\right)-B\left(v\right)e^{-j2\pi \frac{v\left(N+N_{g}\right){\hat{\varepsilon }}_{F}}{N}}\right|}^{2}-\kappa \left(v\right){\bar{\sigma }}_{\omega }^{2}\left(n_{\Lambda }\right).$$

**Figure 5:**
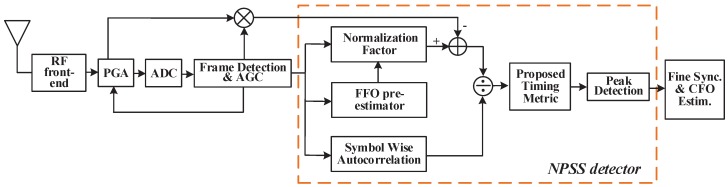
Block diagram of the proposed NPSS detector structure.

After the calculation of $\alpha_{SW}\left(\tau \right)$ and $\beta_{NE}\left(\tau \right)$, the proposed timing metric $\lambda_{prop}\left(\tau \right)$ can be obtained with the ratio of the former two parameters. The implementation structure is shown in Figure 5.

When the coarse timing synchronization is completed and the location of NPSS is decided, the FFO obtained by the FFO pre-estimator can be used as the estimated value to compensate FFO. Besides, as we have mentioned in Section 4, the NB-IoT multi-path channels have considerable RMS-DS, particularly in ETU channel. In addition, such a large RMS-DS may not only bring the uncertainty of ToA estimation, but also introduce inter-symbol interference (ISI). To counteract these two problems, the fine timing should be completed in time domain joint with IFO estimation. Based on the above considerations, a joint ML fine timing and IFO estimation method following [8] is applied. The cost function of the joint fine timing and IFO estimation can be expressed as

(32) $$\Psi \left(\tau_{f},\varepsilon_{I}\right)=\sum_{n=\tau_{c}-\upsilon }^{N_{NPSS}+\tau_{c}+\upsilon -1}\sum_{m=3}^{M}r_{5,r_{f}}^{\left(m\right)}\left(n\right)s_{5,r_{f}}^{*(m)}\left(n-\tau_{c}+\upsilon \right)e^{\frac{j2\pi \varepsilon_{I}\left(n-\tau_{c}+\upsilon \right)}{N}},$$

where $\tau_{f}$ and $\tau_{c}$ represent the fine timing offset and coarse timing offset respectively, and $N_{NPSS}$ denotes the number of samples within one NPSS duration. To counteract the RMS-DS introduced by multi-path channel, whose typical value is 991 ns of ETU, the fine timing gird search range [−υ,υ] must be two times greater than it. Besides, since the NB-IoT frequency offset range is [−25.5 KHz, 25.5 KHz] in theory, the gird search range of IFO should be [−1,1]. Finally, the correct fine timing and IFO can be obtained by finding the maximum value of absolute value of the two-dimensional grid search of the above cost function.

### 5.2. NSSS Detection and Cell ID Identification

According to the discussion in Section 1, the non-coherent partial correlation may be a preferable solution for NB-IoT NSSS detection. However, the 504 cell IDs are differentiated by four complementary sequences, and this means that any partial correlation applied to the NSSS will increase the probability of error detection. What is worse is that NB-IoT actually needs to deal with 2016 different candidate sequences because NSSS is constructed by both cell IDs and frame numbers. To identify the transmitted NSSS sequence, the number of matched filters (MF) should theoretically be equal to the candidate sequences. By using the complementary property, Abdelmohsen Ali and Walaa Hamouda reduce the scale of the MF bank to one-quarter of direct implementation [8]. However, the power and hardware resources occupied by a massive amount of complex multipliers are still intolerable for an NB-IoT terminal. In our design, we combine the four complementary sequences and frame number terms of Equation (2) into reconstructed complementary sequences, and then the frame number and cell ID are obtained by maximum likelihood estimation.

**Figure 6:**
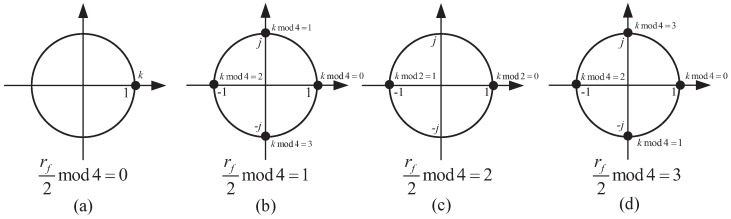
Diagram of generation of the four complementary sequences based on frame number.

We define the frame number term $e^{-j2\pi \theta_{f}k}$ as $T_{r_{f}}$. By inspecting Table 2, it can be observed that $T_{r_{f}}$ is a cyclic variable of $r_{f}$ and *k*. Figure 6 gives the detailed illustration.

Because the four cyclic sequences represented by Figure 6a–d contain only real units and imaginary units, we combine the four cyclic sequences and complementary sequences to form 16 new complementary sequences which only contain ±1 and ±i. In this way, three-quarters of multiplications used by [8] are saved. On the other hand, as NSSS only locates in even radio frames and the frame number is still unknown, two detection statistics should be calculated simultaneously. The detection window of each statistic is (2W − 1) radio frames, so the entire detection window is 2W.

(33) $$B_{\left(1\right)}\left(u\right)=\sum_{w=1}^{W}\sum_{m=3}^{M}\sum_{k=1}^{12}{\tilde{R}}_{9,r_{f}+2\left(w-1\right)}^{\left(m\right)}\left(k\right)\tilde{b}\left(m,k,u\right),$$

(34) $$B_{\left(2\right)}\left(u\right)=\sum_{w=1}^{W}\sum_{m=3}^{M}\sum_{k=1}^{12}{\tilde{R}}_{9,r_{f}+2w-1}^{\left(m\right)}\left(k\right)\tilde{b}\left(m,k,u\right),$$

where $\tilde{R}$ is the results after applying the 16 complementary sequences as code cover of the received signal *R*. It is important to note that during the cross correlation process, different complementary sequences are used with the variation of radio frame number. $\tilde{b}\left(m,k,u\right)$ represents the part of b(n) without reconstructed complementary sequences, and $n^{\prime }=k+12\left(m-3\right)-1$. Then, the cell ID and frame number can be jointly estimated by

(35) $$\left\{{\hat{N}}_{ID}^{Ncell},{\hat{r}}_{f}\right\}=\underset{u}{\arg\max}\left\{\left|B_{\left(1\right)}\left(u\right)\right|,\left|B_{\left(2\right)}\left(u\right)\right|\right\}.$$

The number of multiplications of this method is 16.6K, which is one-quarter of [8] and one-sixteenth of [33].

### 5.3. Brief Summary of Cell Search

**Figure 7:**
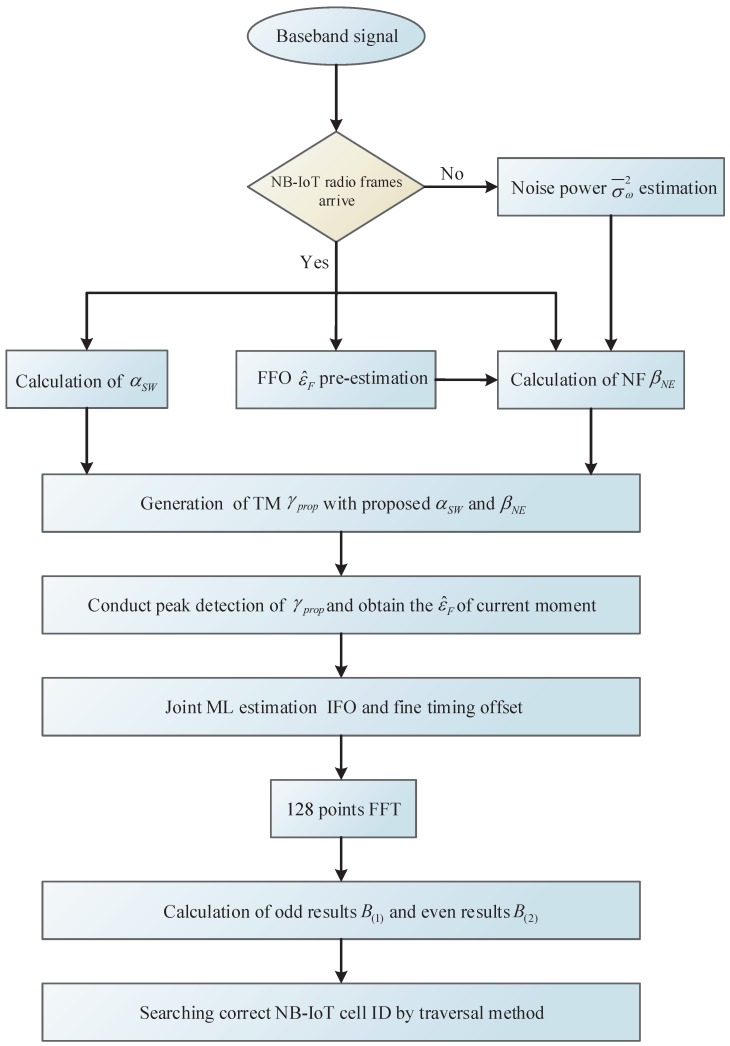
Algorithm flowchart of the proposed cell search scheme.

The whole cell search procedure has been discussed adequately in the two subsections above, and we will briefly summarize it here. We have proposed a noise power estimator and a FFO pre-estimator to improve the performance of the NPSS detector with low SNR and large frequency offset. IFO and fine timing joint estimation is conducted in the time domain with an existing ML cross-correlation solution. Then, we promote a divide-and-conquer cell ID identification method. To make the whole process clearer, a cell search algorithm flowchart is given in Figure 7.

## 6. Frequency Tracking

As mentioned before, frequency tracking of LTE is conducted with the assistance of RS as auxiliary data. However, because of the narrow bandwidth, more pilot signals should be used by NB-IoT for tracking frequency. The 40 ms UTG demands that NPSS, NSSS and NPBCH be regenerated and applied for residual frequency offset estimation [31]. The received frequency domain signal is defined in Equation (8) as $R_{p,f_{f}}^{\left(m\right)}\left(k\right)$ and the stored local data is $S_{p,f_{f}}^{\left(m\right)}\left(k\right)$. Firstly, we investigate two existing methods. Then, our solution is presented in detail and the advantages are also discussed.

### 6.1. Least Square Method

To make use of the subcarrier without identical signals to estimate the frequency offset, one efficient way is to calculate the CFRs to eliminate the effect of multi-path channels. A two-step scheme is presented in [31]. Firstly, the CFR of each pilot symbol is obtained by least square estimation, which is carried out by

(36) $${\hat{H}}_{p,f_{f}}^{\left(m\right)}\left(k\right)=R_{p,f_{f}}^{\left(m\right)}\left(k\right){\left(S_{p,f_{f}}^{\left(m\right)}\left(k\right)\right)}^{-1}.$$

Then, under the assumption that the CFRs are constant within the correlation distance *D*, we divide the CFRs into two vectors. They are $H_{1}=[{\hat{H}}_{p,f_{f}}^{\left(m_{1}\right)}\left(k\right)\cdots {\hat{H}}_{p,f_{f}}^{\left(m_{i}\right)}\left(k\right)\cdots {\hat{H}}_{p,f_{f}}^{\left(m_{I}\right)}\left(k\right){]}^{T}$ and $H_{2}=[{\hat{H}}_{p,f_{f}}^{\left(m_{1}+D\right)}\left(k\right)\cdots {\hat{H}}_{p,f_{f}}^{\left(m_{i}+D\right)}\left(k\right)\cdots {\hat{H}}_{p,f_{f}}^{\left(m_{I}+D\right)}\left(k\right){]}^{T}$. The RFO is estimated by

(37) $${\hat{\varepsilon }}_{RFO}=\frac{N}{2\pi D(N+N_{g})}∠\left(H_{1}^{H}H_{2}\right),$$

where *I* represents the number of pilot correlation pairs and ${\left(\cdot \right)}^{H}$ is Hermitian transposition.

### 6.2. Cross Correlation Assistant Method

The authors of [28] use the cross-correlation between received signals and located signals to eliminate the phase difference of pilots. They adopt the RS of LTE as pilots, and the RS symbols from the same antenna port are spaced by *D* symbols. Additionally, the CFR of symbols in one subcarrier is considered constant during *D* symbols. Then, this estimator can be accomplished by

(38) $${\hat{\varepsilon }}_{RFO}=\frac{N}{2\pi D(N+N_{g})}∠\left(\sum_{i=1}^{I}\left(R_{p,f_{f}}^{\left(m_{i}\right)}\left(k\right)S_{p,f_{f}}^{*(m_{i})}\left(k\right)R_{p,f_{f}}^{*(m_{i+D})}\left(k\right)S_{p,f_{f}}^{\left(m_{i+D}\right)}\left(k\right)\right)\right).$$

### 6.3. Proposed Adaptive RFO Tracking Method

The above methods have a common drawback, which is that too many multiplications cause an excessive burden on the NB-IoT terminals. As the frequency tracking process is a continuous process during the whole communication transmission, we propose an adaptive RFO tracking solution which can effectively reduce the number of multiplications. Considering the features of NB-IoT, our tracking algorithm can be implemented with the following two steps:

(1) Under the condition that initial synchronization and cell search have been accomplished, and NPSS, NSSS and NPBCH have been regenerated. Then, we define the symbol space vector as $D=[d_{1},d_{2}\cdots d_{J}]$, where *J* denotes the number of available correlation pairs. Then, all the possible correlation pairs are computed beforehand.
  (39) $$U_{i}^{j}=S_{p,f_{f}}^{*(m_{i})}\left(k\right)S_{p,f_{f}}^{\left(m_{i+d_{j}}\right)}\left(k\right).$$
  These precalculated signals can be represented by $U_{i}^{j}$, and $I_{j}$ denotes the number of correlation pairs when the correlation space is *j* symbols.
(2) Once RFO tracking needs to be conducted, the following estimator is applied.
  (40) $${\hat{\varepsilon }}_{RFO}=\frac{N}{2\pi D(N+N_{g})}∠\left(\sum_{j=1}^{j_{0}}\sum_{i=1}^{I_{j}}\left(R_{p,f_{f}}^{\left(m_{i}\right)}\left(k\right)R_{p,f_{f}}^{*(m_{i+d_{j}})}\left(k\right)U_{i}^{j}\right)\right).$$
  The adaptive factor is $1\leq j_{0}\leq J$, which can be adjusted according to the current SNR.

By means of preprocessing, the proposed method can save nearly one-third multiplications compared with [28]. Besides, the adjustable number of correlation pairs can maintain a balance between performance and power consumption.

## 7. Simulation Results

In this section, all the schemes proposed in this paper are simulated, and we also analyze the results.

### 7.1. Simulation Environment

To evaluate the effectiveness of the considered cell search and frequency tracking methods, we carry out the in-band downlink NB-IoT system. There are two antenna ports at the transmitter and one antenna port at the receiver in in-band mode. As for the communication scenarios, the AWGN, ETU and EPA channels are investigated. Because NB-IoT terminals usually have low mobility, the Doppler drift is set as 1 Hz, which means that the coherence time is far greater than one radio frame. The crystal oscillator error is ±20 ppm, so the frequency offset should be ±25.5 KHz under the assumption of 900 MHz carry frequency and ±7.5 KHz raster channel offset. The effective bandwidth and subcarrier spacing are 180 KHz and 15 KHz, respectively. At the receiver, the processing frequency is 1.92 MHz and a 128-point FFT converts the time domain signals to frequency domain signals. Under this condition, N=128 and $N_{g}=10$ when the symbol index is 0 or 7, and $N_{g}=9$ when the symbol index is another value. Moreover, since the NB-IoT terminals should achieve different coverage, we investigate the normal coverage for SNR ≤−6 dB and enhanced coverage for −15 dB ≤SNR≤−6 dB. When we verify the method of frequency tracking, the time window is set as UTG. Additionally, the proposed solution can also achieve continuous frequency tracking with slight modification.

### 7.2. Performance Assessment

**Figure 8:**
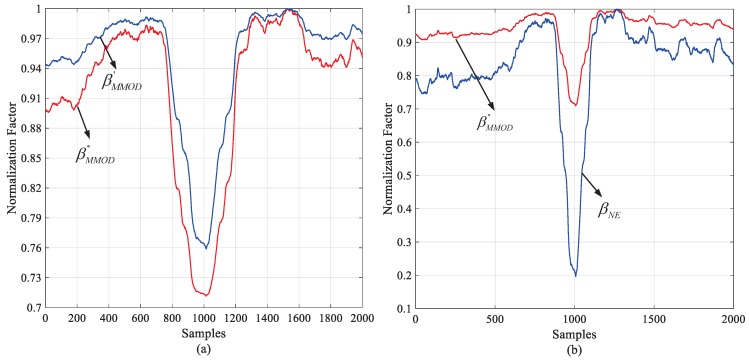
Performance of the proposed normalization factors: (**a**) Comparison of modified MOD vs. frequency offset-compensated MOD normalization factors; (**b**) Comparison of frequency offset-compensated MOD vs. frequency offset-compensated and noise-eliminated MOD normalization factors.

Figure 8 illustrates the performance of normalization factors under the condition of the AWGN channel and −10 dB SNR. All the curves are normalized to the maximum of each. $\beta_{MMOD}^{\prime }$ and $\beta_{MMOD}^{\prime \prime }$ are compared in Figure 8a, and the latter gives a deeper notch. As shown in Equations (23) and (28), $\beta_{MMOD}^{\prime \prime }$ is the FFO elimination version of $\beta_{MMOD}^{\prime }$. For this reason, when the correct timing is absent, the attached FFO compensation makes no contribution. When the correct timing is present, the pre-estimated FFO helps eliminate primary phase error between the two differential components. Figure 8b indicates the promotion of noise elimination. The relative notch after noise elimination factor $\beta_{NE}$ is more obvious than $\beta_{MMOD}^{\prime \prime }$. It is easy to deduce that this promotion will be more significant with lower SNR.

**Figure 9:**
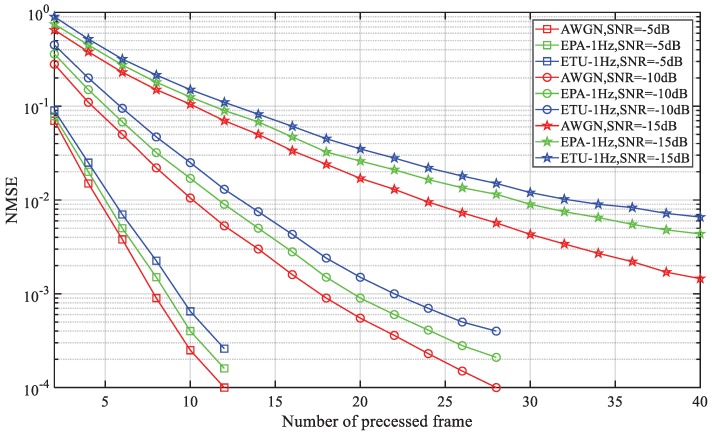
Performance of the FFO pre-estimator with different channel and SNR conditions.

The normalized mean square error (NMSE) of pre-estimated FFO is shown in Figure 9. This simulation is conducted with different channels and SNRs. The initial FFO is set as 7.5 KHz, so $\varepsilon_{F}$ is 0.5. According to Equation (22) and considering the worst condition of V= 10, the residual FFO should be less than $5\times 10^{-2}$. The result shows that even under extended coverage SNR, this condition can be fulfilled within 18 radio frames. Even the theory of this FFO pre-estimator is similar to [7], and increases computation to some extent. The pre-estimated FFO can make the differential normalization effective, which is proved invalid under large-frequency offset in [12]. Additionally, a typical residual FFO is 50 Hz, which means that $6.7\times 10^{-3}$ should be accomplished. We can see that this target is achieved within 40 radio frames, which outperforms [7].

**Figure 10:**
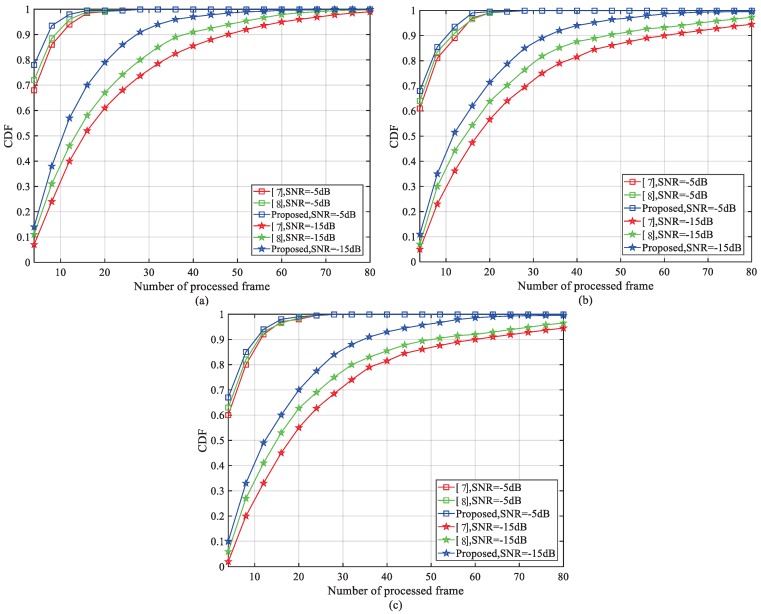
Comparison of cumulative distribution function (CDF) for NPSS detection vs. the number of processed frames under normal coverage and extended coverage: (**a**) AWGN; (**b**) ETU-1Hz; (**c**) EPA-1Hz.

Figure 10 presents the NPSS cumulative probability distribution (CDF) vs. the number of processed frames. The simulation SNR is −5 dB and −15 dB. The simulation channel of Figure 10a–c is AWGN, ETU-1Hz and EPA-1Hz, respectively. The operation frequency of the proposed method and [8] is 1.92 MHz, and the solution in [7] uses 240 KHz frequency to reduce power consumption. From the simulation results, the proposed method outperforms the others. In particular, when the NB-IoT terminals work with extended coverage SNR = −15 dB, our method can achieve a target CDF of 0.9 with ten more radio frames less than [7,8]. When SNR = −5 dB, the proposed method can also provide considerable performance gain. That is because the proposed NF uses the pre-estimated FFO and noise power to accelerate the detection process. However, as nothing can be complete in both respects, we have declared that in order to achieve rapid NPSS detection, acceptable hardware resources and computation complexity are sacrificed in Section 1. In particular, when compared with the solution in [7], the working frequency of our method is eight times its size, and the noise power estimator, FFO pre-estimator, and normalization factor almost quadruple the computation complexity. Nevertheless, there are two aspects that support that such a compromise can save more power than is consumed. One is that the initial NPSS detection only needs to be conducted at the start of each transmission. Then, we can see from Figure 10 that in order to achieve 0.9 CDF, our method can save more than 15 NB-IoT frames (150 ms), and during such a long time, the RF transceiver will consume much more power than baseband digital circuits.

**Figure 11:**
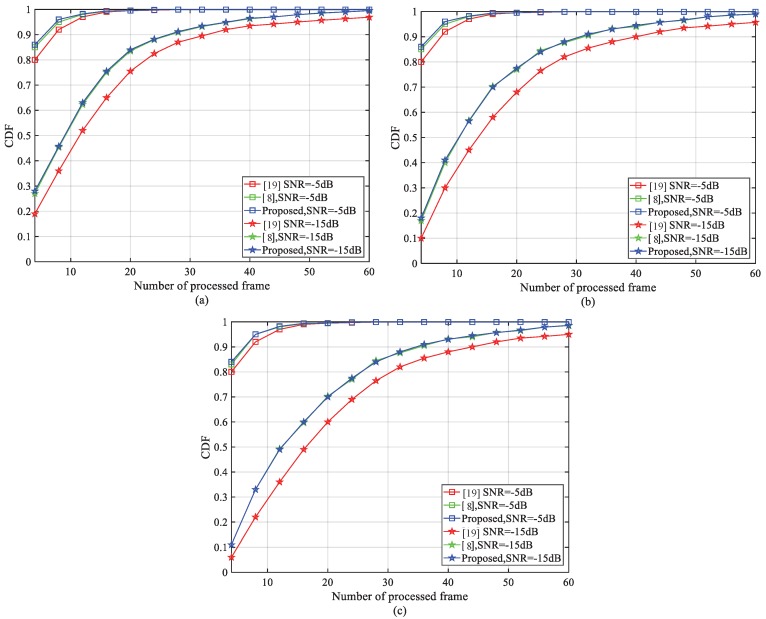
Comparison of the cumulative distribution function (CDF) for cell ID detection vs. the number of processed frames under normal coverage and extended coverage. (**a**) AWGN, (**b**) ETU-1Hz, (**c**) EPA-1Hz.

To investigate the capability of cell ID detection, the CDF under different numbers of processed frames and SNRs is illustrated in Figure 11. The results are obtained by using the AWGN, ETU-1Hz and EPA-1Hz channel models. Figure 11a–c indicate that a target CDF of 0.9 can be achieved within 36 radio frames under −15 dB SNR. The solution proposed in [8] can also achieve similar performance. However, since we convert the frame number part into four complex complementary sequences, the number of base NSSS vectors is reduced from 2016 to 126. Moreover, as the frame number is unknown, two identical detectors work simultaneously to generate $B_{\left(1\right)}$ and $B_{\left(2\right)}$. By applying the aforementioned operations, much lower computation complexity and latency is obtained compared with [8].

**Figure 12:**
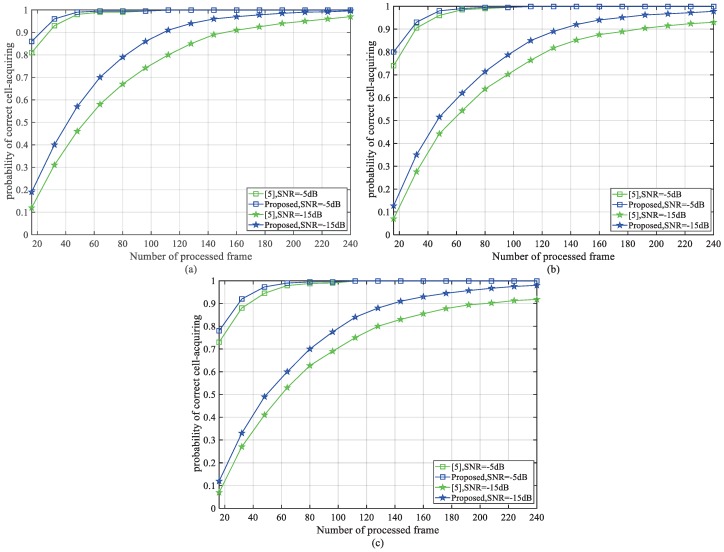
Comparison of probability of correct cell-acquiring vs. the number of processed frames under normal coverage and extended coverage. (**a**) AWGN, (**b**) ETU-1Hz, (**c**) EPA-1Hz.

To give a more direct reflection of our contribution on reducing the initial processing time, Figure 12 displays the probability of correct cell-acquiring vs. the number of processed frames. The simulation SNR is −5 dB and −15 dB. The simulation channel of Figure 12a–c is AWGN, ETU-1Hz and EPA-1Hz separately. Compared with the latest work [8], our method significantly reduces the number of processed frames under different channel conditions, and the performance gets better with lower SNR. It means that the power which is originally consumed within these frames can be saved by applying our method.

**Figure 13:**
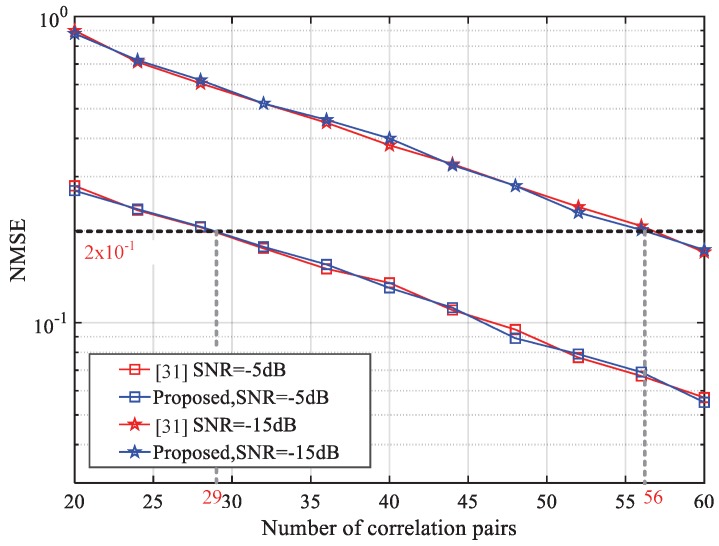
Comparison of frequency tracking performance vs. different numbers of correlation pairs under normal coverage and extended coverage.

In the frequency tracking process, our method is the low complexity version of the cross-correlation assistant method. Figure 13 compares the performance of the proposed method and least square method. The SNR condition is −5 dB and −15 dB and we investigate the AWGN channel model. The tracking time window is set as 40 ms which is defined in [7] for UTG and the initial residual frequency error is 200 Hz. The NMSE of residual frequency error vs. $j_{0}$ is shown in Figure 11. We see that under the same conditions, the two methods have almost the same performance, and the target recovery accuracy of 40 Hz can be achieved by using 56 correlation pairs under extended coverage. However, the proposed method uses one multiplier which replaces the two dividers used by the cross-correlation assistant method to save hardware resources. Furthermore, as the SNR may be different in different transmission processes, we can adjust the number of correlation pairs $j_{0}$ to make a trade-off between computation complexity and performance.

## 8. Conclusions and Future Work

In this paper, we investigated the novel cell search and frequency tracking approaches for NB-IoT to make economic and green IoT networks. Because of the narrow bandwidth, too much time is taken to conduct the initial cell search. We propose an NPSS detector with FFO pre-estimation and noise elimination. Thanks to the application of the proposed NF, the NPSS detector outperforms previous works. As for cell ID detection in the frequency domain, we adopt a divide-and-conquer method to minimize the number of correlation sequences, which reduces the amount of computation by nearly three-quarters compared with the latest scheme. To make the frequency tracking process more efficient, some preprocessing is done before the residual frequency offset estimation. Furthermore, the adjustable number of correlation pairs helps to make a trade-off between the performance and computation complexity. All of the above proposed schemes can contribute to construct a low complexity and robust green NB-IoT network.

In the future, we will continuously explore both rapid and low complexity solutions to improve existing methods. The ultra-extended coverage scenario, such as SNR = −20 dB, will also be investigated. Under such a condition, many existing cell search schemes will be invalid, and if not, the execution time will be seriously lengthened, which implies a burden on power consumption. What is worse is that if frequency tracking cannot be completed within the UTG, the uplink will be unable to work properly. In addition to the above, we will investigate the problem of NB-IoT positioning with the assistance of NPRS. Furthermore, we will also focus on constructing a complete NB-IoT downlink communication link. The transmission of all the downlink physical channels will be supported.

## Figures and Tables

**Table 1:** Code cover sequence.

c(0)	c(1)	c(2)	c(3)	c(4)	c(5)	c(6)	c(7)	c(8)	c(9)	c(10)
1	1	1	1	−1	−1	1	1	1	−1	1

**Table 2:** Derivation of the parameters in NSSS.

Parameters	Formulation
$n^{\prime }$	nmod131
*m*	nmod128
*u*	$N_{ID}^{Ncell}\;\mathrm{mod}\;126+3$
*q*	$\left\lfloor \frac{N_{ID}^{Ncell}}{126}\right\rfloor$
$\theta_{f}$	$\frac{r_{f}}{8}\;\mathrm{mod}\;4$
